# Cytochrome *c* oxidase subunit 1 gene as a DNA barcode for discriminating *Trypanosoma cruzi* DTUs and closely related species

**DOI:** 10.1186/s13071-017-2457-1

**Published:** 2017-10-16

**Authors:** Marina Silva Rodrigues, Karina Alessandra Morelli, Ana Maria Jansen

**Affiliations:** 1 Laboratory of Trypanosomatid Biology, Oswaldo Cruz Institute, Fiocruz, Rio de Janeiro, Brazil; 2 Department of Ecology, Institute of Biology Roberto Alcantara Gomes, State University of Rio de Janeiro, Rio de Janeiro, Brazil

**Keywords:** Cytochrome *c* oxidase subunit 1, *Trypanosoma cruzi*, Discrete typing units, Glucose-6-phosphate isomerase, Barcoding, Subgenus *Trypanosoma* (*Schizotrypanum*)

## Abstract

**Background:**

The DNA barcoding system using the cytochrome *c* oxidase subunit 1 mitochondrial gene (*cox*1 or *COI*) is highly efficient for discriminating vertebrate and invertebrate species. In the present study, we examined the suitability of *cox*1 as a marker for *Trypanosoma cruzi* identification from other closely related species*.* Additionally, we combined the sequences of *cox*1 and the nuclear gene glucose-6-phosphate isomerase (*GPI*) to evaluate the occurrence of mitochondrial introgression and the presence of hybrid genotypes.

**Methods:**

Sixty-two isolates of *Trypanosoma* spp. obtained from five of the six Brazilian biomes (Amazon Forest, Atlantic Forest, Caatinga, Cerrado and Pantanal) were sequenced for *cox*1 and *GPI* gene fragments. Phylogenetic trees were reconstructed using neighbor-joining, maximum likelihood, parsimony and Bayesian inference methods. Molecular species delimitation was evaluated through pairwise intraspecific and interspecific distances, Automatic Barcode Gap Discovery, single-rate Poisson Tree Processes and multi-rate Poisson Tree Processes.

**Results:**

Both *cox*1 and *GPI* genes recognized and differentiated *T. cruzi*, *Trypanosoma cruzi marinkellei*, *Trypanosoma dionisii* and *Trypanosoma rangeli*. *Cox*1 discriminated Tcbat, TcI, TcII, TcIII and TcIV. Additionally, TcV and TcVI were identified as a single group. *Cox*1 also demonstrated diversity in the discrete typing units (DTUs) TcI, TcII and TcIII and in *T. c. marinkellei* and *T. rangeli*. *Cox*1 and *GPI* demonstrated TcI and TcII as the most genetically distant branches, and the position of the other *T. cruzi* DTUs differed according to the molecular marker. The tree reconstructed with concatenated *cox*1 and *GPI* sequences confirmed the separation of the subgenus *Trypanosoma* (*Schizotrypanum*) sp. and the *T. cruzi* DTUs TcI, TcII, TcIII and TcIV. The evaluation of single nucleotide polymorphisms (SNPs) was informative for DTU differentiation using both genes. In the *cox*1 analysis, one SNP differentiated heterozygous hybrids from TcIV sequences. In the *GPI* analysis one SNP discriminated Tcbat from TcI, while another SNP distinguished TcI from TcIII.

**Conclusions:**

DNA barcoding using the *cox*1 gene is a reliable tool to distinguish *T. cruzi* from *T. c. marinkellei*, *T. dionisii* and *T. rangeli* and identify the main *T. cruzi* genotypes.

**Electronic supplementary material:**

The online version of this article (10.1186/s13071-017-2457-1) contains supplementary material, which is available to authorized users.

## Background

How many species are there on Earth? Estimations suggest that approximately 90% of species remain undescribed [1]. The identification and classification of biodiversity is a practice that has always fascinated humankind. The Greek philosopher Aristotle (4th century BC) developed the first classification system, which was used for nearly 2000 years [2]. In the 1700s, Carl Linnaeus [3] developed the concept of binomial nomenclature using Latin, which was the language of educated men at his time. Binomial nomenclature is a standard method for scientists that speak different languages to classify living things to clearly communicate their discoveries. Recently, DNA sequences have been applied in the classification of life forms. However, different methods and DNA regions have been used to compare the same taxonomic groups, frequently leading to conflicting results.

In the search for a simple method to identify and compare species, Hebert et al. [4] proposed DNA barcoding, a new system of species identification using the cytochrome *c* oxidase subunit 1 mitochondrial gene (*cox*1 or *COI*) as a standardized single molecular marker for the classification of animal species. One of the requirements of the DNA barcoding approach is that species identification is associated with a voucher belonging to a curated biological collection, enabling follow up and a strategy for corroborating species identification [5]. Until recently, national barcode networks have been established in 11 countries, including Brazil, which uses the Brazilian Barcode of Life (BrBOL) [6].

In addition to the identification of known and new species, barcoding with the *cox*1 gene is suggested as a standard for cryptic taxa discovery, association of different life stages of the same species and wildlife conservation genetics [5]. *Cox*1 appears to have a better phylogenetic signal than the other mitochondrial genes [7]. Some authors argue that the evolution of the *cox*1 gene is sufficiently rapid to discriminate between closely related species and investigate intraspecific diversity [4, 8]. The *cox*1 region is highly efficient for discriminating vertebrate and invertebrate species [5, 9, 10], but is not suited for plants and some fungal species [4, 11, 12]. The use of *cox*1 for identifying protozoa and other unicellular organisms is still in its initial stage but has been demonstrated as a promising barcode marker for dinoflagellates, *Paramecium* sp., Nemertea [13–15]. Moreover, joint analyses of nuclear and mitochondrial markers may be necessary to distinguish species [1].

Therefore, the small subunit ribosomal DNA (SSU rDNA) has been proposed as a first step for a protist barcode, followed by the use of a second marker specific for each group [6]. To confirm a gene as a barcode marker, it is essential to calculate the “barcoding gap”, that is, the gap between the maximum intraspecific and minimum interspecific genetic distances that establish the limits between species [16]. Several molecular targets for trypanosomatid identification have previously been used: internal transcribed spacer (ITS) [17], mini-exon [18], glucose-6-phosphate isomerase (*GPI*) [19–21], cytochrome *b* (*cytb*) [22, 23], multilocus sequencing typing (MLST) [24], the variable regions V7 V8 of SSU rDNA, and glyceraldehyde-3-phosphate dehydrogenase (*gGAPDH*) genes [22, 25, 26]. However, there is no consistency concerning which DNA region to use as a trypanosomatid barcode, and none of these markers were evaluated as a universal marker for biodiversity analysis.

With the emergence of molecular techniques with higher analytical power, an increasing number of new species of *Trypanosoma* have been reported [26, 27] and it is becoming increasingly clear that there is a high diversity of *Trypanosoma* spp. within the subgenus *Schizotrypanum* of *Trypanosoma*. The extreme morphological similarity and phylogenetic proximity of these species, and the use of tools with lower analytical power, resulted in the isolation of numerous species of *T.* (*Schizotrypanum*) from the wild transmission cycle remaining only at the generic level or wrongly diagnosed as *Trypanosoma cruzi*, highlighting the importance of a universal method to characterize *T. cruzi*, its subpopulations and representatives of the subgenus *Schizotrypanum*.

**Fig. 1:**
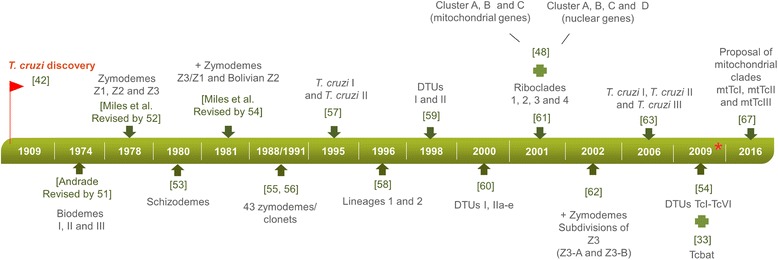
Designations of *T. cruzi* subpopulations through time and according to the method employed. The numbers represent dates of publication. The red star indicates current consensus of *T. cruzi* nomenclature and subdivisions

*Trypanosoma cruzi* (Trypanosomatida: Trypanosomatidae) is a successful parasite that is capable of establishing an extracellular cycle in dozens of triatomine species (Hemiptera: Reduvidae) and infecting almost every cellular type from more than 100 mammal species distributed from the south of the USA to the south of Latin America [28, 29]. Although described as one taxon, *T. cruzi* has a remarkable genetic heterogeneity that was already recognized by the very first authors who researched it [30–32]. The *T. cruzi* population structure has been classified as clonal or, at least, primarily clonal [32, 33] and hybridization events have also been observed, suggesting that genetic recombination occurs in this group [33, 34]. Moreover, the extent to which these hybridization events are responsible for the enormous heterogeneity of this parasite remains a controversial issue [19, 35–37]. Several techniques, (biological, biochemical and molecular) applied to define *T. cruzi* subpopulations have led to different designations (Fig. 1) [25, 30, 35, 38–51]. Presently, *T. cruzi* subpopulations are assembled in six discrete typing units (DTUs) - named TcI, TcII, TcIII, TcIV, TcV, TcVI [29] - and a genotype called Tcbat, initially described as being associated with Chiroptera [25], although it was previously isolated from a child [52] and from mummified human tissue [53]. However, Barnabé et al. [51] recently proposed the subdivision of *T. cruzi* into three mitochondrial clades (mtTcI, mtTcII and mtTcIII) based on the analysis of the nucleotide sequences available in the GenBank database, showing that the classification of *T. cruzi* subpopulations remains a debatable issue.

In the present study, we tested *cox*1 as a DNA barcode to identify *T. cruzi* from other closely related species belonging to the subgenus *Schizotrypanum* and to examine the genetic diversity within *T. cruzi* and its DTUs to further understand the ecology of the species of *T.* (*Schizotrypanum*). Additionally, we evaluated *cox*1 as a target to also identify *Trypanosoma* (*Tejeraia*) *rangeli*, as this trypanosome shares the geographical distribution, vectors and mammal hosts of *T. cruzi* and is also included in the *T. cruzi* clade [54]*.* To evaluate the occurrence of mitochondrial introgression events and the presence of hybrid genotypes we tested a combination of *cox*1 (uniparental inheritance) and the nuclear gene glucose-6-phosphate isomerase (*GPI*) (biparental inheritance). *GPI* is one of the genes sequenced for an extensive number of *T. cruzi* isolates distributed over several geographic regions [51], enabling a comparison of the sequences generated in the present study. Thus, the precise identification of these *Trypanosoma* species is of the utmost importance. In addition, the present study will enhance the amount of nucleotide sequences available for comparison, since the GenBank database still lacks a broader deposit on trypanosomatid sequences for the *cox*1 gene.

## Methods

### Samples

**Fig. 2:**
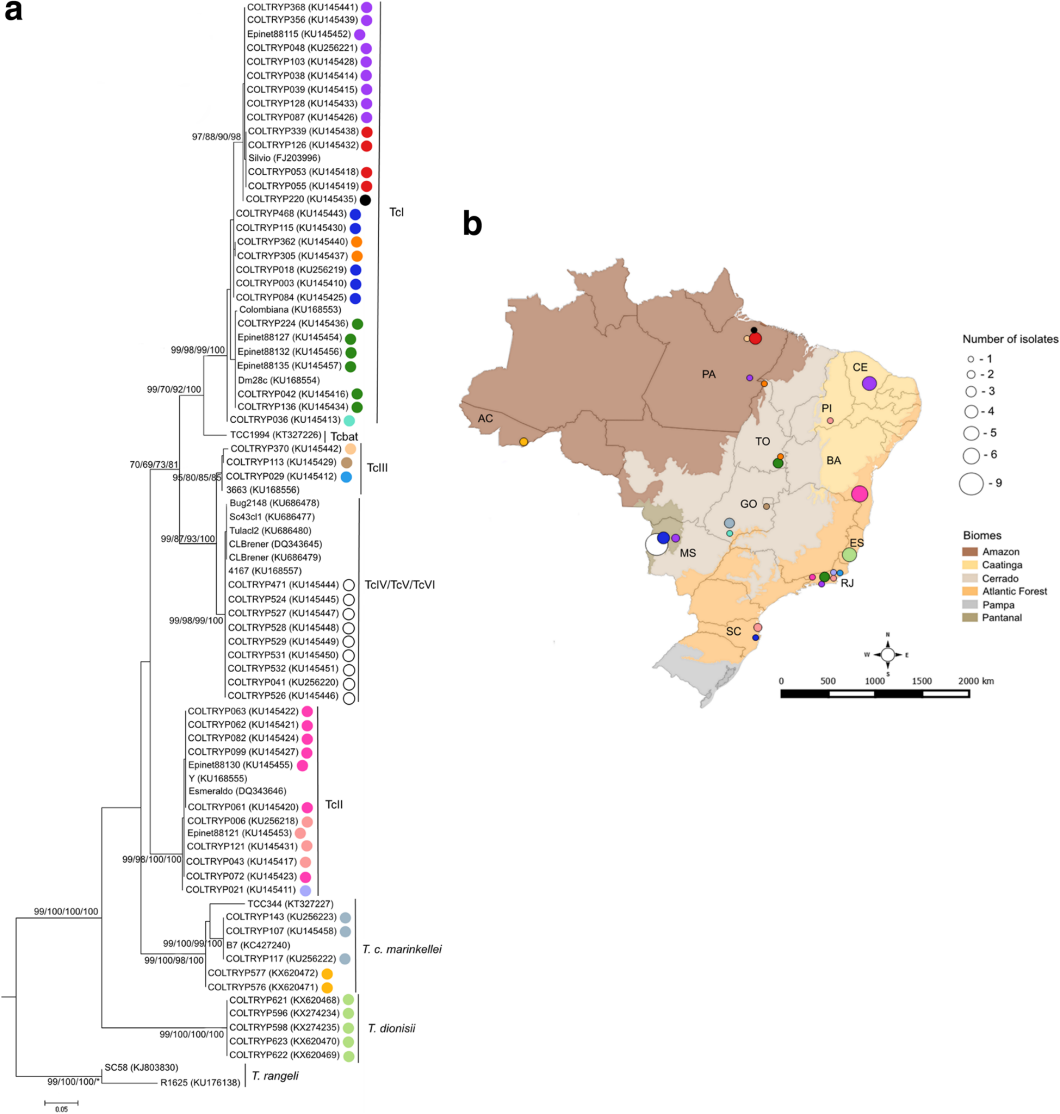
Phylogenetic tree based on *cox*1 and the geographical origin of the isolates under study. **a** The *cox*1 gene differentiates *T. cruzi* DTUs TcI, TcII, TcIII and TcIV, Tcbat, *T. c. marinkellei*, *T. dionisii* and *T. rangeli*. The heterozygous hybrids TcV and TcVI cannot be differentiated and were placed into the same cluster as TcIV. The numbers at the nodes correspond to NJ, ML, MP and BI support values, respectively (only values >60 are shown). The scale-bar shows the number of nucleotide substitutions per site. The different haplotypes correspond to the diversity observed in *cox*1 sequences and are represented by colors in the tree. **b** The map represents the distribution of the characterized isolates among Brazilian biomes. Each colored circle indicates a different haplotype. Circle size represents the number of haplotypes. *Abbreviations*: Brazilian states: AC, Acre; BA, Bahia; CE, Ceara; ES, Espírito Santo; GO, Goiás; MS, Mato Grosso do Sul; PA, Pará; PI, Piauí; RJ, Rio de Janeiro; SC, Santa Catarina; TO, Tocantins

The present study included 62 isolates of *Trypanosoma* spp. obtained from 16 different genera of free-ranging wild mammals and from triatomines of the genera *Rhodnius* and *Triatoma*, distributed in five of the six Brazilian biomes (Amazon Forest, Atlantic Forest, Caatinga, Cerrado and Pantanal) (Table 1 Fig. 2b). Isolates were cryopreserved and deposited in the Coleção de *Trypanosoma* de Mamíferos Silvestres, Domésticos e Vetores, Fiocruz - COLTRYP (Oswaldo Cruz Foundation, Rio de Janeiro - RJ/Brazil) in previous studies. Details on animal capture and parasite culture methods are described elsewhere [55–57]. After thawing, the cells were sown in axenic culture media (NNN and LIT). When the cultures reached the exponential growth phase, DNA was extracted using the phenol-chloroform method, as described elsewhere [58]. The *T. dionisii* samples used in the present study were molecularly identified using 18S (SSU) and gGAPDH sequences and subsequently deposited in COLTRYP.

**Table 1:** Molecular identification, geographical and host origin of the COLTRYP isolates and reference stocks under study

Strain code	Host or Vector	State/Biome	Lineage	Hap (*cox*1)^a^	GenBank accession number	
					*cox*1	*GPI*
*T. cruzi*						
TCC 1994	*Myotis levis*	SP/Brazil	Tcbat	hap9	KT327226^b^	KT327312^b^
COLTRYP 038	*Rattus rattus*	CE/Caatinga	TcI	hap3	KU145414	KT390200
COLTRYP 039	*Rattus rattus*	CE/Caatinga	TcI	hap3	KU145415	KT390201
COLTRYP 048	*Didelphis albiventris*	CE/Caatinga	TcI	hap3	KU256221	KU256227
COLTRYP 087	*Didelphis albiventris*	CE/Caatinga	TcI	hap3	KU145426	KT390212
COLTRYP 128	*Didelphis albiventris*	CE/Caatinga	TcI	hap3	KU145433	KT390219
COLTRYP 018	*Thylamys macrurus*	MS/Pantanal	TcI	hap1	KU256219	KU256225
COLTRYP 084	*Oecomys* sp*.*	MS/Pantanal	TcI	hap1	KU145425	KT390211
COLTRYP 103	*Monodelphis domestica*	MS/Pantanal	TcI	hap3	KU145428	KT390214
COLTRYP 115	*Thylamys macrurus*	MS/Pantanal	TcI	hap1	KU145430	KT390216
COLTRYP 368	*Nasua nasua*	MS/Pantanal	TcI	hap3	KU145441	KT390227
COLTRYP 468	*Oecomys mamorae*	MS/Pantanal	TcI	hap1	KU145443	KT390229
COLTRYP 053	*Rhodnius pictipes*	PA/Amazon	TcI	hap2	KU145418	KT390204
COLTRYP 055	*Didelphis marsupialis*	PA/Amazon	TcI	hap2	KU145419	KT390205
COLTRYP 126	*Didelphis marsupialis*	PA/Amazon	TcI	hap2	KU145432	KT390218
COLTRYP 220	*Didelphis marsupialis*	PA/Amazon	TcI	hap5	KU145435	KT390221
COLTRYP 339	*Rhodnius pictipes*	PA/Amazon	TcI	hap2	KU145438	KT390224
Epinet 88,115	*Rhodnius robustus*	PA/Amazon	TcI	hap3	KU145452	KT390238
COLTRYP 356	*Akodon cursor*	RJ/Atlantic Forest	TcI	hap3	KU145439	KT390225
Epinet 88,127	*Rhodnius* sp.	RJ/Atlantic Forest	TcI	hap4	KU145454	KT390240
Epinet 88,132	*Philander frenatus*	RJ/Atlantic Forest	TcI	hap4	KU145456	KT390242
Epinet 88,135	*Philander frenatus*	RJ/Atlantic Forest	TcI	hap4	KU145457	KT390243
COLTRYP 003	*Didelphis aurita*	SC/Atlantic Forest	TcI	hap1	KU145410	KT390196
COLTRYP 036	*Didelphis albiventris*	GO/Cerrado	TcI	hap8	KU145413	KT390199
COLTRYP 042	*Desmodus rotundus*	TO/Cerrado	TcI	hap4	KU145416	KT390202
COLTRYP 136	*Phyllostomus albicola*	TO/Cerrado	TcI	hap4	KU145434	KT390220
COLTRYP 224	*Carollia perspicillata*	TO/Cerrado	TcI	hap4	KU145436	KT390222
COLTRYP 305	*Philander opossum*	TO/Cerrado	TcI	hap6	KU145437	KT390223
COLTRYP 362	*Gracilinanus* sp.	TO/Cerrado	TcI	hap6	KU145440	KT390226
Colombiana	*Homo sapiens*	Colombia	TcI	hap7	KU168553	KU168558
Dm28c	*Didelphis marsupialis*	Venezuela	TcI	hap4	KU168554	KU168559
Silvio	*Homo sapiens*	PA/Amazon	TcI	hap2	FJ203996^b^	
OPS21cl11	*Homo sapiens*	Venezuela	TcI			AY484472^b^
COLTRYP 061	*Leontopithecus chrysomelas*	BA/Atlantic Forest	TcII	hap11	KU145420	KT390206
COLTRYP 062	*Leontopithecus chrysomelas*	BA/Atlantic Forest	TcII	hap11	KU145421	KT390207
COLTRYP 063	*Leontopithecus chrysomelas*	BA/Atlantic Forest	TcII	hap11	KU145422	KT390208
COLTRYP 072	*Leontopithecus chrysomelas*	BA/Atlantic Forest	TcII	hap11	KU145423	KT390209
COLTRYP 081	*Leontopithecus chrysomelas*	BA/Atlantic Forest	TcII	hap11	KU145424	KT390210
COLTRYP 099	*Leontopithecus chrysomelas*	BA/Atlantic Forest	TcII	hap11	KU145427	KT390213
COLTRYP 006	*Thrichomys apereoides*	PI/Caatinga	TcII	hap10	KU256218	KU256224
COLTRYP 021	*Leontopithecus rosalia*	RJ/Atlantic Forest	TcII	hap12	KU145411	KT390197
COLTRYP 121	*Leontopithecus rosalia*	RJ/Atlantic Forest	TcII	hap10	KU145431	KT390217
Epinet 88,130	*Philander frenatus*	RJ/Atlantic Forest	TcII	hap11	KU145455	KT390241
COLTRYP 043	*Triatoma tibiamaculata*	SC/Atlantic Forest	TcII	hap10	KU145417	KT390203
Epinet 88,121	*Triatoma tibiamaculata*	SC/Atlantic Forest	TcII	hap10	KU145453	KT390239
Y	*Homo sapiens*	SP/Brazil	TcII	hap11	KU168555	KU168560
Esmeraldo	*Homo sapiens*	BA/Brazil	TcII	hap11	DQ343646^b^	
Tu18cl2	*Triatoma infestans*	Bolivia	TcII			AY484477^b^
COLTRYP 113	*Monodelphis domestica*	GO/Cerrado	TcIII	hap14	KU145429	KT390215
COLTRYP 370	*Rhodnius pictipes*	PA/Amazon	TcIII	hap15	KU145442	KT390228
COLTRYP 029	*Galictis vittata*	RJ/Atlantic Forest	TcIII	hap13	KU145412	KT390198
3663	*Panstrongylus geniculatus*	AM/Brazil	TcIII	hap13	KU168556	KU168561
M6241 cl6	*Homo sapiens*	PA/Brazil	TcIII			AY484478^b^
COLTRYP 041	*Thrichomys pachyurus*	MS/Pantanal	TcIV	hap16	KU256220	KU256226
COLTRYP 471	*Oecomys mamorae*	MS/Pantanal	TcIV	hap16	KU145444	KT390230
COLTRYP 524	*Triatoma* sp.	MS/Pantanal	TcIV	hap16	KU145445	KT390231
COLTRYP 526	*Triatoma* sp.	MS/Pantanal	TcIV	hap16	KU145446	KT390232
COLTRYP 527	*Triatoma* sp.	MS/Pantanal	TcIV	hap16	KU145447	KT390233
COLTRYP 528	*Triatoma* sp.	MS/Pantanal	TcIV	hap16	KU145448	KT390234
COLTRYP 529	*Triatoma* sp.	MS/Pantanal	TcIV	hap16	KU145449	KT390235
COLTRYP 531	*Triatoma* sp.	MS/Pantanal	TcIV	hap16	KU145450	KT390236
COLTRYP 532	*Triatoma* sp.	MS/Pantanal	TcIV	hap16	KU145451	KT390237
4167	*Rhodnius brethesi*	AM/Brazil	TcIV	hap16	KU168557	KU168562
CANIIIcl1	*Homo sapiens*	PA/Brazil	TcIV			AY484474^b^
Sc43cl1	*Triatoma infestans*	Bolivia	TcV	hap17	KU686477	KU686481
Bug2148	*Triatoma infestans*	RS/Brazil	TcV	hap17	KU686478	KU686482
CLBrener	*Triatoma infestans*	SP/Brazil	TcVI	hap17	KU686479	KU686483
Tulacl2	*Homo sapiens*	Chile	TcVI	hap17	KU686480	KU686484
CLBrener	*Triatoma infestans*	SP/Brazil	TcVI	hap17	DQ343645^b^	
Bug2148_1	*Triatoma infestans*	RS/Brazil	TcV			HQ452737^b^
Bug2148_2	*Triatoma infestans*	RS/Brazil	TcV			HQ452738^b^
CLBrener_1	*Triatoma infestans*	SP/Brazil	TcVI			HQ452739^b^
CLBrener_2	*Triatoma infestans*	SP/Brazil	TcVI			HQ452740^b^
*T. c. marinkellei*						
COLTRYP 107	*Phyllostomus discolor*	GO/Cerrado		hap18	KU145458	KT390244
COLTRYP 117	*Phyllostomus discolor*	GO/Cerrado		hap18	KU256222	KU256228
COLTRYP 143	*Phyllostomus discolor*	GO/Cerrado		hap18	KU256223	KU256229
COLTRYP 576	*Phyllostomus hastatus*	AC/Amazon		hap19	KX620471	KX620476
COLTRYP 577	*Phyllostomus hastatus*	AC/Amazon		hap19	KX620472	KX620477
B7	*Phyllostomus discolor*	BA/Brazil		hap18	KC427240^b^	AY484485^b^
TCC 344	*Carollia perspicillata*	RO/Brazil		hap19	KT327227^b^	KT327313^b^
*T. dionisii*						
COLTRYP 596	*Anoura geoffroyi*	ES/Atlantic Forest		hap21	KX274234	KX274236
COLTRYP 598	*Carollia* sp.	ES/Atlantic Forest		hap21	KX274235	KX274237
COLTRYP 621	*Anoura geoffroyi*	ES/Atlantic Forest		hap21	KX620468	KX620473
COLTRYP 622	*Carollia* sp.	ES/Atlantic Forest		hap21	KX620469	KX620474
COLTRYP 623	*Carollia* sp.	ES/Atlantic Forest		hap21	KX620470	KX620475
*T. rangeli*						
R1625	*Homo sapiens*	El Salvador		hap23	KU176138	KU176137
RGB	*Canis familiaris*	Colombia				AY484486^b^
SC58	*Echimys dasythrix*	SC/Brazil		hap24	KJ803830^b^	

^a^Hap (*cox*1): haplotype inferred for *cox*1 in DnaSP v5.10.01

^b^Sequences retrieved from GenBank

*Abbreviations*: Brazilian states: *AC* Acre, *AM* Amazonas, *BA* Bahia, *CE* Ceará, *ES* Espírito Santo, *GO* Goiás, *MS* Mato Grosso do Sul, *PA* Pará, *PI* Piauí, *RJ* Rio de Janeiro, *RO* Rondônia, *RS* Rio Grande do Sul, *SC* Santa Catarina, *SP* São Paulo, *TO* Tocantins

Nine *T. cruzi* references were also genotyped and used as DTU standards. Colombiana (COLPROT 004), Dm28c (COLPROT 010), Y (COLPROT 106), 3663 (COLPROT 608), 4167 (COLPROT 607) DNA samples obtained from the Protozoa Collection - COLPROT (FIOCRUZ, Rio de Janeiro, Brazil); Sc43cl1, Bug2148cl1, CLBrener and Tulacl2 DNA samples were a kind gift from Dr. Michael Miles from the London School of Hygiene & Tropical Medicine (London, UK). The *T. rangeli* lineage R1625 DNA sample (COLPROT 002) was obtained from COLPROT (Table 1). In addition, *cox*1 and *GPI* sequences were retrieved from the GenBank database and used as references (Table 1).

### PCR and sequencing

A fragment of the mitochondrial gene *cox*1 was amplified using a set of forward (5′-CCA WAC AAC AAA CAT ATG ATG CTG C-3′) and reverse (5′-TCC HGA TAT GGT WTT KCC YCG-3′) primers. Polymerase chain reaction (PCR) was conducted in a 50 μl final reaction volume containing 2.5 mM MgCl_2_ (Invitrogen, Carlsbad, California, USA), 0.25 mM of each nucleotide (Thermo Scientific, Waltham, Massachusetts, USA), 0.25 mM of each primer (IDT, Coralville, Iowa, USA), 1.0 U of Platinum Taq DNA polymerase (Invitrogen), and 50 ng of DNA template. The amplification was performed using a Veriti 96-Well Thermal Cycler (Applied Biosystems, Foster City, California, USA) with the following cycle conditions: denaturation at 95 °C for 3 min; followed by 35 cycles at 95 °C for 1 min, 54 °C for 1 min, and 72 °C for 1 min; and a final elongation step at 72 °C for 10 min. Nucleotide sequences were also determined using a fragment of the nuclear gene *GPI*. The primers and cycling conditions are described elsewhere [20]. The PCR products were separated on 1.5% agarose gels and stained with GelRed (Biotium Inc., Fremont, California, USA). The fragments were purified using the Wizard Genomic DNA Purification Kit, according to manufacturer’s instructions (Promega, Madison, Wisconsin, USA), and direct sequencing of both strands of DNA was performed with BigDye Terminator v3.1 Cycle Sequencing Kit (Applied Biosystems) using an ABI 3730 DNA sequencer available at the RPT01A/FIOCRUZ sequencing facilities.

### Data analysis

The sequences were manually edited using Geneious software version 8.1.6. (Biomatters, Auckland, New Zealand) and aligned using the CLUSTAL X version 2.1 multiple alignment program [59]. All sequences were translated to confirm the absence of premature stop codons. All sequences generated were deposited in the GenBank database (Table 1).

The heterozygous hybrid lineages (TcV and TcVI) amplified for *GPI* were subjected to haplotype reconstruction using the PHASE algorithm implemented in DnaSP v5.10.01 [60].

The neighbor-joining (NJ) method and Kimura 2-parameters (K2P) model were applied for both *cox*1 and *GPI* genes according to the barcode approach [4]. NJ analyses were performed with MEGA version 6 [61]. For each node, bootstrap percentages (BP) were computed after 1000 resamplings.

The maximum likelihood (ML) method was also applied to each topology. The model of nucleotide substitution that best fitted the *cox*1 data was the Hasegawa-Kishino-Yano’s model (HKY), with a gamma-distributed rate (Γ). For *GPI*, the best-fit model was the Tamura-Nei model, with a gamma-distributed rate. These models were selected using the Akaike Information Criterion corrected for small samples (AICc) approach implemented in the program jModelTest [62]. ML analyses were performed using PhyML 3.0 [63]. For each node, BP were computed after 1000 resamplings.

Maximum Parsimony (MP) analyses were performed using PAUP* 4.0b10 [64]. For the tree search and bootstrap we used a heuristic search with 100 random sequence addition replicates through tree bisection and reconnection (TBR) branch-swapping algorithm. Bayesian inference (BI) was run in MrBayes v3.2.6 [65] with a general time reversible model with gamma-distributed rate variation across sites and a proportion of invariable sites (GTR + Γ + I). The runs converged after 1,000,000 generations, by sampling every 100th generation and discarding the first 25% of the trees as ‘burn-in’. *Cox*1 and *GPI* sequences were concatenated in SequenceMatrix 1.8 [66] and submitted to NJ, ML, MP and BI analysis as described above.

The number of haplotypes, nucleotide diversity (π) and haplotype diversity (Hd) were calculated for both genes, except for Tcbat, which had a single sequence available in GenBank. The analyses were run in DnaSP v5.10.01 [60].

Molecular species delimitation was evaluated using distance-based methods and coalescent-based models. Distance-based analyses included the pairwise intraspecific and interspecific distances calculated using MEGA version 6 [61] and the Automatic Barcode Gap Discovery (ABGD) method, which detects a gap in the distribution of pairwise distances and uses this information to partition the sequences into groups of hypothetical species [67]. ABGD analysis was conducted in the web version: Jukes Cantor, K2P and p distances were calculated, and the remaining parameters were used as default [67]. Coalescent-based analysis included the single-rate Poisson Tree Processes (PTP) model [68], which considers that every species evolved at the same rate, and the multi-rate Poisson Tree Processes (mPTP) [69], which assumes a different evolution rate for each species. PTP and mPTP analyses were conducted using the web version of this software [69].

## Results

*Cox*1 and *GPI* gene fragments were successfully amplified for a panel of 62 *Trypanosoma* spp. isolates and ten reference strains (Table 1). All sequences were translated to amino acids and compared to *cox*1 and *GPI* proteins. No indels (insertions/deletions) or stop codons were detected. No pseudo genes or contaminants were observed.

### Phylogenetic tree reconstruction using *cox*1 as barcode

The clusters observed in the *cox*1 trees were the same for all methods tested, indicating that these groups are robust and do not depend on the evolutionary methods selected (Fig. 2a). *Cox*1 discriminated species belonging to the subgenus *Schizotrypanum* and *T. cruzi* DTUs. TcI and Tcbat were closely related but clearly constitute two different DTUs with a statistical support of 99, 70, 92 and 100 in NJ, ML, MP and BI analysis, respectively (Fig. 2a)*.* TcIII and TcIV sequences were separated into two different clusters with bootstrap values of 99, 87, 93 and 100 in NJ, ML, MP and BI analysis, respectively. For the heterozygous hybrid lineages, TcV and TcVI formed an indistinguishable group in the same cluster as TcIV.

### Phylogenetic tree reconstruction using *GPI*

**Fig. 3:**
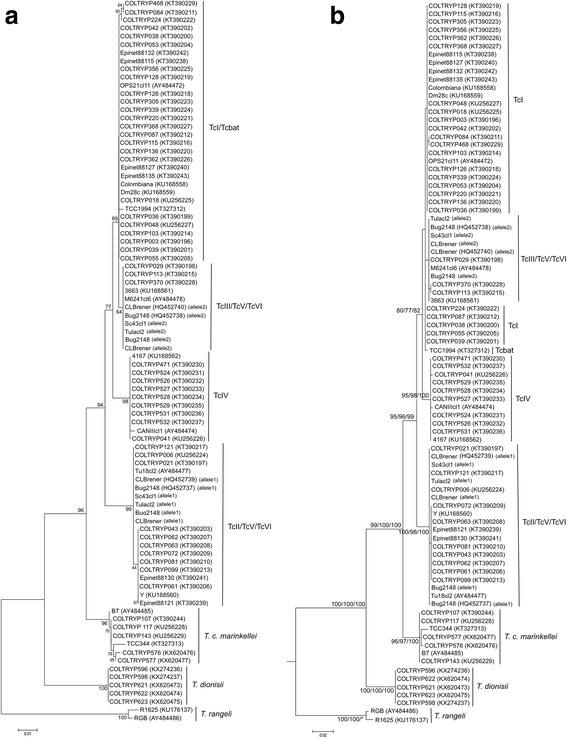
Phylogenetic tree based on nuclear gene *GPI*. **a** Tree inferred with neighbor-joining method and Kimura-2-parameter model. *GPI* recognizes and differentiates *T. cruzi* DTUs TcI, TcII, TcIII and TcIV, *T. c. marinkellei*, *T. dionisii* and *T. rangeli*. One allele of TcV and TcVI sequences cluster with TcII, and the other allele clusters with TcIII. Tcbat was placed in the same cluster as TcI. **b** The tree inferred from maximum likelihood, parsimony and Bayesian inference. *T. cruzi*, *T. c. marinkellei*, *T. dionisii* and *T. rangeli* are clearly separated from each other. DTUs TcI and TcII are the most genetically divergent. Tcbat, TcI and TcIII fall in the same cluster. One allele of TcV and TcVI clustered with TcII, and the other allele clustered with the group comprising TcI, TcIII and Tcbat. The numbers at the nodes correspond to ML, MP and BI support values, respectively (only values >60 are shown). The scale-bar shows the number of nucleotide substitutions per site

Both *GPI* and *cox*1 helped recognize *Schizotrypanum* species, but not *T. cruzi* DTUs. Depending on the method used for the phylogenetic tree reconstruction there was a slightly different topology. Tcbat and TcI clustered together and could not be differentiated in the tree (Fig. 3). In NJ analysis TcIII constituted a separate DTU close to TcI (Fig. 3a). However, with ML, MP and BI methods TcI, Tcbat and TcIII clustered together (bootstrap of 80, 77 and 82, respectively) (Fig. 3b). *GPI* sequences generated for TcV and TcVI presented electropherograms with double peaks (i.e. with two bases at the same position) and were submitted to haplotype reconstruction prior to use in the final alignments and tree reconstructions. This analysis resulted in two sequences for each hybrid sample corresponding to alleles. One allele was closer to TcII, and the other allele was closer to TcIII (Fig. 3).

The geographical distribution of the trypanosomatid isolates under study is represented in Fig. 2b. Both *cox*1 and *GPI* sequences demonstrated the differences between *T. cruzi*, *T. c. marinkellei*, *T. dionisii* and *T. rangeli* and also, to some degree, *T. cruzi* lineages. *Cox*1 and *GPI* phylogenies equally demonstrated that TcI and TcII are the most genetically distant branches, but showed differences concerning the positions of the DTUs TcIII, TcIV, TcV, TcVI and Tcbat in the phylogenetic trees. The mitochondrial gene *cox*1 may be a better discriminator of *T. cruzi* lineages, identifying five DTUs and TcV/TcVI as a single group (Fig. 2a). Additionally, these differences between mitochondrial and nuclear tree topologies, no incongruence was observed in DTU assignment (Table 1), and mitochondrial introgression events were absent in the present sample set.

### Identification of *T. cruzi* DTUs through single nucleotide polymorphisms (SNPs)

Some *T. cruzi* sequences were not clearly assigned to a DTU based solely on information from the trees. In the *cox*1 tree, TcIV and TcV/TcVI sequences were arranged in the same cluster (Fig. 2a), whereas in *GPI* analyses, TcI, TcIII and Tcbat separation was blurred (Fig. 3). Therefore, the multiple sequence alignment of *cox*1 and *GPI* data was considered for the evaluation of single nucleotide polymorphisms (SNPs) using *T. cruzi* sequences only. These polymorphisms were informative to DTU differentiation for both genes.

In the *cox*1 gene fragment analysis, we identified 84 polymorphic sites. We observed a single nucleotide polymorphism (SNP) that differentiates the heterozygous hybrids from TcIV sequences. A T (thymine) was present at position 1264 of the *cox*1 gene in all ten TcIV sequences analyzed, whereas TcV and TcVI sequences display a C (cytosine) at the same site. No polymorphism differentiating TcV from TcVI was observed (Additional file 1: Figure S1).

In the *GPI* analysis we identified 20 polymorphic sites concerning all *T. cruzi* sequences. A thymine at position 315 separates Tcbat from TcI (cytosine) and one guanine to adenine change separates TcI from TcIII at position 396 of the gene (Additional file 2: Figure S2). No polymorphism discriminating TcV from TcVI sequences was observed.

### Phylogenetic tree reconstruction using concatenated data

The concatenation of *cox*1 and *GPI* gene fragments confirmed, with robust statistical support values, the separation of species belonging to the subgenus *Schizotrypanum* and the *T. cruzi* DTUs.

**Fig. 4:**
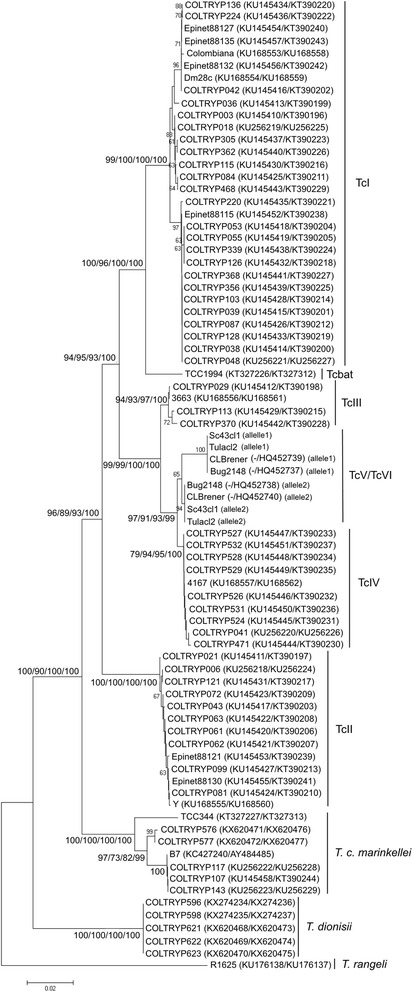
Phylogenetic tree based on the concatenation of *cox*1 and *GPI* sequences. The concatenated data show a clear separation between *T. cruzi* DTUs TcI, TcII, TcIII and TcIV, Tcbat, *T. c. marinkellei*, *T. dionisii* and *T. rangeli*. The heterozygous hybrids TcV and TcVI were not differentiated from each other. The numbers at the nodes correspond to NJ, ML, MP and BI support values, respectively (only values >60 are shown). The scale-bar shows the number of nucleotide substitutions per site

TcI, TcII, TcIII and TcIV sequences constituted clearly separated clades. In addition, concatenated data supported Tcbat as a sister clade to TcI. The heterozygous hybrids TcV and TcVI could not be differentiated and formed a cluster separate from TcIV. The topologies observed in the trees were the same for the four methods tested (NJ, ML, MP and BI) and were supported by values above 80 in the main branches (Fig. 4).

### Genetic diversity evaluated with *cox*1 and *GPI* genes

Although the *cox*1 gene did not distinguish TcV from TcVI, this gene demonstrated diversity in *T. cruzi* DTUs TcI, TcII and TcIII and *T. c. marinkellei* and *T. rangeli* (Additional file 3: Table S1, Fig. 2b). *GPI* sequences also displayed distinct haplotypes in *T. c. marinkellei* and *T. rangeli*, but lower intra-DTU diversity. The correlation between haplotype and geographical area or host species was not evident.

In the *cox*1 analysis, TcI was demonstrated as the most diverse DTU with the highest nucleotide diversity and haplotype diversity of all DTUs, followed by TcIII and TcII. The TcI isolates and reference strains in the present study were distributed in eight haplotypes throughout five Brazilian biomes (Fig. 2b) and six different host orders (Carnivora, Chiroptera, Didelphimorphia, Hemiptera, Primates and Rodentia) (Table 1). In the Amazon, we observed the highest number of different TcI haplotypes in the Para state (hap 2, 3 and 5). Haplotypes 2 and 5 were observed only in the Para State, while haplotype 3 was widely distributed and detected in four different biomes (Amazon, Atlantic Forest, Caatinga and Pantanal). Additionally, in Para, we observed two different haplotypes in three TcI isolates derived from *Didelphis marsupialis* (Table 1). Thus, establishing a correlation between the TcI haplotype and location or host species would be premature. Substantial genetic diversity was also observed in TcIII sequences. In four TcIII isolates derived from *Monodelphis domestica* (Cerrado biome), *Galictis vittata* (Atlantic Forest), *Rhodnius pictipes* (Amazon biome) and a reference strain from *Panstrongylus geniculatus* (Amazon biome), we identified three different haplotypes (Table 1, Fig. 2b). These TcII isolates were divided into three haplotypes and originated from primates, a marsupial and triatomines captured in the Atlantic Forest and from a rodent captured in the Caatinga biome. The reference strains were isolated from humans in the Atlantic Forest. The three TcII haplotypes were distributed in the state of Rio de Janeiro, isolated from a *Philander frenatus* and two *Leontopithecus rosalia* (Table 1). Genetic diversity was not detected among the TcIV, TcV and TcVI isolates. Diversity could not be evaluated for Tcbat, reflecting the unique sequence available for this DTU.

The seven *T. c. marinkellei* isolates displayed three haplotypes (Additional file 3: Table S1, Fig. 2b). One haplotype was formed by isolates originated from *P. discolor* from the Goiás State (Cerrado) and the Bahia state (Atlantic Forest); another haplotype comprised isolates originated from *P. hastatus* from the Acre State (Amazon biome); and a third group was formed by the reference strain TCC 344, isolated from *C. perspicillata* (Amazon) (Table 1, Fig. 2a). Despite the low number of isolates, an apparent correlation between haplotype and host species was observed.

We compared two *T. rangeli* isolates previously identified as lineages C and D [54, 70, 71]. Sequences generated with *cox*1 exhibited two different haplotypes (Table 1, Additional file 3: Table S1). *Trypanosoma dionisii* sequences showed no diversity. Isolates derived from two different species of phyllostomid bats from the same geographical area and collected during the same field expedition (Table 1, Additional file 3: Table S1).

In the *GPI* analysis, intra-DTU diversity was lower than observed with *cox*1. TcI, TcII, TcIII and TcIV sequences displayed two different haplotypes each. In TcIV, one haplotype was formed by the nine isolates identified herein and the reference strain 4167, while the other haplotype only comprised the reference strain CANIII, which was not available for *cox*1 analysis. No diversity within TcV/TcVI sequences was detected.

The highest diversity in *GPI* sequences was observed in *T. c. marinkellei* with five haplotypes. One haplotype comprised three isolates from the Goiás state (Cerrado) and the other haplotypes corresponded to the other four isolates (Additional file 3: Table S1, Fig. 3). The two *T. rangeli* sequences generated with *GPI* were identified elsewhere as lineage C [71]. However, these sequences displayed distinct haplotypes (Additional file 3: Table S1, Fig. 3). No diversity within *T. dionisii* sequences was observed. No correlation between haplotype, host or geographical location could be established.

### Barcoding gap and species delimitation with *cox*1 and *GPI* sequences

Based on the analysis of the barcode gaps we assessed and compared the efficiency of *cox*1 and *GPI* for the identification of trypanosomatids. In *cox*1 the mean genetic distance between *T. cruzi* sequences and *T. c. marinkellei* was 13%. The mean intraspecific divergence for *T. cruzi* was 7.6%, a value lower than the mean interspecific value. However, comparing the minimum and maximum values, we observed an overlap of the genetic distances between *T. cruzi* and *T. c. marinkellei* (10.1–15.3%) and the intraspecific divergence for *T. cruzi* (0–12.7%), indicating the absence of a limit that separates these two subspecies (i.e. absence of a barcoding gap). The divergence between *T. cruzi* and *T. dionisii* limits was 13.0–16.0%, while the divergence between *T. cruzi* and *T. rangeli* was 14.7–21.3%, indicating that the genetic distance separating *T. cruzi*, *T. dionisii* and *T. rangeli* as different species is 0.3 and 2.0%, respectively (Additional file 4: Table S2).

*GPI* was not as discriminative as *cox*1. The mean interspecific divergence between *T. cruzi* sequences and *T. c. marinkellei*, *T. dionisii* and *T. rangeli* was 3.6, 7.8 and 13.7%, respectively.

We observed differences between *T. c. marinkellei* sequences in the trees (Fig. 3) confirmed by an intraspecific distance ranging from 0 to 1.0% (Additional file 5: Table S3). Distance within *T. rangeli* sequences was 0.7% and there was no genetic difference between *T. dionisii* sequences. For *T. cruzi*, the intraspecific genetic distance ranged from 0 to 3.0%, i.e. lower than the mean interspecies values (Table 2). Similar to *cox*1, we observed an overlap of the genetic distances between *T. cruzi* and *T. c. marinkellei* with *GPI*, indicating the absence of a “barcoding gap”.

**Table 2:** *cox*1 and *GPI* sequences division in groups based on ABGD analysis

Substitution model	X^a^	Partition	Prior intraspecific divergence (P)								
			0.059948	0.035938	0.021544	0.012915	0.007743	0.004642	0.002783	0.001668	0.001000
*cox*1											
Jukes Cantor	1.5	Initial	6	6	6	10	10	10	10	10	10
		Recursive			8	11	14	14	14	21	21
K2P^b^	1.5	Initial	6	6	6	11	11	11	11	11	11
		Recursive			8		14	14	14	19	19
p-distance	1.5	Initial		6	6	6	6	6	6	6	6
		Recursive		0	8	9	9	9	11	11	11
*GPI*											
Jukes Cantor	1.5	Initial	2	2	3	3	5	7	3	14	14
		Recursive							7		
K2P^b^	1.5	Initial	2	2	3	3	5	7	3	14	14
		Recursive							7		
p-distance	1.5	Initial	2	2	3	3	5	5	5	7	7
		Recursive									

^a^X, relative gap width

^b^ *K2P* Kimura 2-parameter

*Trypanosoma cruzi* and *T. c. marinkellei* were separated into distinct groups according to ABGD, PTP and mPTP analysis using *cox*1 and *GPI* data. Here, we report the results for the three substitution models and both initial and recursive partitions in the output of ABGD. The results varied from 6 to 21 for *cox*1 depending on the substitution model used, confirming diversity in *T. cruzi* sequences and the separation of *T. cruzi*, *T. c. marinkellei*, *T. rangeli* and *T. dionisii* (Table 2). *Trypanosoma cruzi* sequences were divided into a minimum of three groups in the three models tested. One group corresponded to Tcbat and TcI; another group corresponded only to TcII sequences only; and a third group comprised TcIII, TcIV, TcV and TcVI sequences. A maximum of 13 different groups were observed, with TcI sequences divided into eight different groups, showing the higher intra-DTU diversity of TcI compared to the other DTUs (Additional file 6: Table S4). In all models and partitions, *T*. *dionisii* sequences were arranged in one group.

The number of ABGD groups for *GPI* data varied from 2 to 14 depending on the model applied (Table 2). *Trypanosoma cruzi* sequences were separated into different groups. TcI sequences were grouped together, showing less variability with *GPI*. TcII sequences were divided into two groups: one group with only TcII sequences and another group with TcII, TcV (allele 1) and TcVI (allele 1). TcIII sequences were grouped together with sequences representing the other TcV and TcVI alleles. TcIV sequences were combined in one group, except for the reference strain CANIII, which was placed in a separated group. *Trypanosoma cruzi marinkellei* and *T. rangeli* sequences were divided into groups, reaffirming their diversity (Additional file 7: Table S5), while *T. dionisii* sequences formed one group in all tests (Table 2, Additional file 7: Table S5).

The number of groups recovered by ABGD was higher than the number of species studied. However, this finding confirms the genetic diversity within *T. cruzi* DTUs, *T. c. marinkellei* and *T. rangeli* observed in the phylogenetic trees (Figs. 2a and 3).

The PTP and mPTP models identified, respectively, a total of 10 and 7 putative species in the *cox*1 dataset (Table 3). Four of these putative species were subdivisions of *T. cruzi,* indicating the heterogeneity of this taxon. The PTP model also recognized diversity within *T. c. marinkellei* and *T. rangeli* sequences.

**Table 3:** Number of species according to PTP and mPTP delimitation methods

Gene and taxon	PTP	mPTP
	Number of putative species	
*cox*1		
*T. cruzi*	4	4
*T. c. marinkellei*	3	1
*T. dionisii*	1	1
*T. rangeli*	2	1
Total	10	7
*GPI*		
*T. cruzi*	3	3
*T. c.marinkellei*	1	1
*T. dionisii*	1	1
*T. rangeli*	2	1
Total	7	6

PTP and mPTP provided a similar number of putative species for *GPI* sequences (Table 3). *Trypanosoma cruzi* was divided into three groups, and *T. c. marinkellei* sequences were allocated into one group. The difference between models was observed in the *T. rangeli* sequences, separated into two groups or placed into one group.

In *cox*1 and *GPI* analysis using both models, *T. cruzi, T. c. marinkellei, T. rangeli* and *T. dionisii* were recognized as different species. The diversity of *T. cruzi* was confirmed, and no diversity was observed in *T. dionisii* sequences.

## Discussion

In the present study, the DNA barcoding approach using the *cox*1 gene has been demonstrated to be efficient at recognizing *Trypanosoma* species and their major subpopulations. With *cox*1, we distinguished *T. cruzi* from *T. c. marinkellei*, *T. donisii* and *T.* (*Tejeraia*) *rangeli*, fulfilling the main DNA barcode demands of a short gene fragment that can be sequenced in diverse sample sets and generating comparable sequences that enable the distinction of species from each other [4]. We also generated a library of trypanosome sequences for *cox*1 and *GPI* genes. Each specimen analyzed is linked to an identification number, collection date, country, region and host of origin, geographical coordinates and other information that enable the tracking of the origin of the specimen and ensure the reproducibility of subsequent experiments.

*Trypanosoma cruzi* is currently divided into seven DTUs [29]. Using *cox*1, we identified five *T. cruzi* groups (TcI, TcII, TcIII, TcIV and Tcbat). The DTUs TcI and TcII are consistently shown as the most genetically distant groups, well separated by *cox*1 in all four methods tested (Fig. 2a). This structure has been observed by other authors in trees with high bootstrap support values, sustaining TcI and TcII as the two discernible DTUs, independently of gene or method used [25, 51, 72, 73]. Furthermore, this system showed the potential for separating genetically closer DTUs. We observed Tcbat as a separated cluster within *T. cruzi* and its proximity to TcI, consistent with Marcili et al. [25]. According to other studies using *cytb*, *V7 V8* rRNA and *gGAPDH* genes this relationship is unanimous [26, 51, 74, 75]. However, the data on Tcbat are still limited, as only one sequence was generated with *cox*1 available in GenBank. Our *cox*1 sequences also showed the homozygous hybrids TcIII and TcIV forming distinct groups in all methods tested (Fig. 2a). These DTUs are proposed to have been originated from the genetic exchange between TcI and TcII and evolved separately giving origin to TcIII and TcIV [76, 77]. The genetic proximity between TcIII and TcIV is undeniable, and their separation and position in the phylogenetic trees is altered by the gene and method of inference selected. Based on *cytb* (inferred by MP), *V7 V8*, *gGAPDH*, *GPI* genes and MLST approaches, TcIII and TcIV were identified as two separate DTUs [24–26, 28, 51]. However, in other studies using the *cytb* gene (inferred by neighbor-joining and maximum likelihood), TcIII and TcIV were identified as a single group [34, 51, 77]. TcV and TcVI sequences were indistinguishable and clustered with TcIV (Fig. 2a), consistent with previous *cytb* mitochondrial gene results [25]. In some studies, independently of the molecular marker and phylogenetic method applied, TcV and TcVI were also indistinguishable from each other. However, these hybrids clustered together with TcII or TcIII when analyzed using nuclear markers [25, 26, 34, 78]. In contrast, in studies using 4 to 10 gene fragments and neighbor-joining trees, TcV and TcVI appeared as two distinct DTUs [24, 79]. In the *cox*1 analysis, we observed one SNP that differentiates TcIV sequences from TcV/TcVI (Additional file 1: Figure S1). This nucleotide polymorphism, combined with the phylogenetic tree, was demonstrated as relevant to DTU assignment. However, TcV and TcVI are the less conspicuous lineages, and their separation remains an issue.

*Cox*1 was also suitable to determine diversity within DTUs TcI, TcII and TcIII (Additional file 3: Table S1). The number of sequences classified as TcI and the number of different haplotypes in these DTU sequences were the highest, compared to the other DTUs. The diversity within TcI is consistent with previous studies and may be explained by TcI being a multi-host lineage widely distributed throughout Brazilian biomes, representing the DTU with the largest set of samples analyzed, and consequently, the DTU with the most published studies compared to the other DTUs [20, 51, 52, 80, 81]. The nucleotide and haplotype diversity of the TcII sequences generated using *cox*1 were lower. However, this effect may not reflect the reality, but rather may show subsampling. In the Rio de Janeiro State, we observed one TcII haplotype circulating in a specimen of *L. rosalia* and a different haplotype in another specimen of *L. rosalia*. This observed diversity may reflect primates captured in different years and the changes in TcII haplotype circulation in that area. Nevertheless, the same host can harbor different haplotypes from the same parasite, and one haplotype can prevail over another in different moments of isolation. Diversity within TcII has previously been demonstrated through the sequencing of the glycoprotein 72 gene (*gp72*) and showed that this DTU has a higher distribution range than previously considered [56]. The high haplotype diversity observed in TcIII could result from overestimation, since we identified three different haplotypes in the four sequences analyzed, belonging to isolates from different Brazilian regions. However, this scenario shows TcIII distributed in a wide geographical range, infecting marsupials, carnivores and triatomines (Table 1). This finding clearly indicates that the richness within TcIII, and its dispersion is yet to be explored. Diversity in TcIII has previously been observed based on *V7 V8*, *cytb*, *GPI*, MLST approaches, but no correlation with geographical area or host species was evident [24, 25, 28]. In the present study, TcIV samples were isolated from triatomines and rodents from the same geographical area (Table 1). This aspect might explain the observation of only one haplotype in TcIV sequences. However, isolates from rodents were collected 11 years before the parasites isolated from the intestinal content of triatomines. Thus, we propose that TcIV haplotype circulation in the Pantanal area was at least equally predominant throughout more than a decade. However, the TcIV reference was isolated from a triatomine in the Amazon region (Table 1) and had the same haplotype as the Pantanal isolates. This finding could reflect the conservation of the *cox*1 region in TcIV. A correlation between TcIV diversity and the geographical region has been suggested by other studies based on mitochondrial genes *cytb* and cytochrome *c* oxidase subunit 2-NADH dehydrogenase subunit 1 (*cox*2-*nad*1) [28, 34, 77].

The *cox*1 tree topologies, independently of the method applied, showed *T. c. marinkellei* as a sister clade to the monophyletic clade formed by all *T. cruzi* DTUs (Fig. 2a); we also observed genetic diversity within *T. c. marinkellei* (Additional file 3: Table S1). Even with the characterization of a low number of isolates, the samples were separated into two groups, and a sequence retrieved from GenBank was positioned in a third group (Fig. 2a). Heterogeneity within *T. c. marinkellei* has previously been reported [75, 82, 83]. Subdivision into two major groups (T.c.m.I and II) and a potential third group (lineage Z) was proposed using multilocus enzyme electrophoresis (MLEE) and random amplified polymorphic DNA (RAPD). No association with a host or geographical distribution was confirmed [82]. This lack of evidence for an association and the different markers used prevented the comparison of these data.

We also observed genetic differences between *T. rangeli* reference sequences R1625 and SC58 classified, respectively, as lineage C and lineage D [54, 70]. Previous studies have proposed the subdivision of *T. rangeli* in five lineages (A-E), based on spliced leader and SSU rDNA [54, 70, 71]. Even with only two sequences, we suggest that *cox*1 can distinguish different *T. rangeli* lineages and is a promising tool for use in species identification.

In the present study, we showed the first *T. dionisii* sequences for the *cox*1 gene. The nucleotide sequences were deposited in GenBank, contributing to the enhancement of the barcode public library for *Trypanosoma* species. We did not observe diversity among these sequences (Fig. 2a, Additional file 3: Table S1), likely because the samples were collected from bats of the same area. No subdivisions in groups or subpopulations have been proposed for *T. dionisii* until recently. Although potential diversity within this species can be observed in *cytb* and 18S (SSU) phylogenetic trees, these data were not reported [84].

The concomitant analysis of the mitochondrial gene *cox*1 and the nuclear gene *GPI* enable the confirmation of the absence of mitochondrial introgression events in the sample set. The frequency of this genetic phenomenon is unknown and has primarily been observed in heterozygous hybrid DTUs TcV and TcVI, where uniparental inheritance of maxicircle kDNA is the rule [28, 36, 56, 85]. *Cox*1 has limitations and does not work as a single barcode in all situations [1], and since *T. cruzi* possesses heterozygous hybrid lineages and mitochondrial introgression events have previously been reported, we proposed this *cox*1-*GPI* barcoding system. Additionally, the concatenated analysis of *cox*1 and *GPI* confirmed *T. cruzi* DTU separation (Fig. 4).

Consistent with the *cox*1 results, *GPI* distinguished *T. cruzi* from *T. c. marinkellei*, *T. donisii* and *T.* (*Tejeraia*) *rangeli.* Additionally, with *GPI*, TcI and TcII were separated into two conspicuous groups. This nuclear gene recognizes a lower number of *T. cruzi* groups (Fig. 3) and considerably lower intra-DTU diversity compared to *cox*1 (Additional files 4 and 5: Tables S2 and S3). In contrast to the *cox*1 results, Tcbat and TcI were clustered together. The lower power of resolution to discriminate DTUs and intra-DTU diversity might reflect the fact that *GPI* is a housekeeping nuclear gene, which shows a lower evolution rate than mitochondrial genes [28, 85]. Furthermore, we compared the *GPI* sequences with the single Tcbat sequence available in GenBank and the results may change depending on the number of sequences available. It is likely that a larger set of Tcbat sequences would resolve the incongruence between the mitochondrial and nuclear gene trees. In addition, we also observed differences in DTU placement in the trees according to the method of inference in the *GPI* analyses. TcIII sequences formed a cluster separate from TcI when we applied the neighbor-joining method and clustered together with TcI when maximum likelihood, parsimony and Bayesian inference were used. This effect may reflect the fact that the neighbor-joining method was based on the genetic distance matrix, where a pairwise distance matrix is produced, and the tree is inferred from this matrix; maximum likelihood, parsimony and Bayesian inference were character-based methods of inference where each position of the alignment is analyzed [86]. However, in the *GPI* analysis, we detected SNPs that enable the differentiation of TcI, TcIII and Tcbat (Additional file 2: Figure S2). We observed diversity within *T. c. marinkellei* and generated the first five *T. dionisii* sequences for *GPI*. We could not compare *T. rangeli* sequences generated with *GPI*, as both sequences analyzed belonged to lineage C [54, 70].

Barcoding gap in trypanosomatids is still an unresolved issue. Therefore, there are no parameters or cut-off values available to compare with these results. As expected, we did not observe a barcoding gap between *T. cruzi* and *T. c. marinkellei* because *T. c. marinkellei* is considered a subspecies of *T. cruzi* [87] (Additional files 4 and 5: Tables S2 and S3).

The debate concerning the definition of species will always exist since species are not discrete units, but rather continuous entities.

## Conclusions

The use of partial sequences of *cox*1 and *GPI* genes can clearly identify and separate *T. cruzi* samples from *T. c. marinkellei*, *T. dionisii* and *T. rangeli*. The two-locus barcoding system using *cox*1 and the nuclear gene *GPI* revealed that mitochondrial introgression was absent from the sample set. Additionally, the resolution of *cox*1 at the intraspecific level shows great potential for DTU characterization, separating five DTUs and recognizing the heterozygous hybrids TcV and TcVI as one group different from all the other DTUs; the resolution of *cox*1 at the intraspecific level also demonstrates intra-DTU genetic diversity. Moreover, with *cox*1, we evaluated the diversity within *T. c. marinkellei* sequences and identified two *T. rangeli* lineages. Therefore, the *cox*1 gene is a promising DNA barcode for studying the genus *Trypanosoma* and represents a simple, fast and reliable marker.

## Additional files

Additional file 1: Figure S1. Comparison between TcIV and TcV/TcVI nucleotide sequences generated with the *cox*1 barcode. **a** Alignment of TcIV sequences with TcV and TcVI shows one single nucleotide polymorphism differentiating TcIV samples from the hybrids. **b** Electropherogram confirms the presence of a T (thymine) in TcIV in the same position, showing a C (cytosine) in TcV and TcVI sequences. (TIFF 724 kb)

Additional file 2: Figure S2. Comparison between TcI, Tcbat and TcIII nucleotide sequences generated with *GPI*. **a** Sequence alignment shows one single nucleotide polymorphism differentiating TcI from Tcbat and one polymorphism separating TcI from TcIII sequences. **b** Electropherogram confirms the presence of A (adenine) in TcIII sequences in the same position, showing a G (guanine) in TcI sequences. The Tcbat sequence was retrieved from GenBank and the electropherogram is not publicly available. (TIFF 527 kb)

Additional file 3: Table S1. Number of haplotypes, nucleotide diversity and haplotype diversity of sequences generated with *cox*1 and *GPI*. (DOCX 12 kb)

Additional file 4: Table S2. Inter- and intraspecific genetic distance based on *cox*1 sequences. (DOCX 14 kb)

Additional file 5: Table S3. Inter- and intraspecific genetic distance based on *GPI* sequences. (DOCX 15 kb)

Additional file 6: Table S4. *Cox*1 sequences partition into groups inferred with ABGD, based on Kimura 2-parameters. (DOCX 14 kb)

Additional file 7: Table S5. *GPI* sequences partition into groups inferred with ABGD, based on Kimura 2-parameters. (DOCX 14 kb)

## Abbreviations

ABGD: Automatic Barcode Gap Discovery
BI: Bayesian inference
BrBOL: Brazilian Barcode of Life
COLPROT: Protozoa Collection
COLTRYP: Trypanosomatid collection from wild and domestic mammals and vectors
*cox*1: cytochrome *c* oxidase subunit 1 gene
*cox*2-*nad*1: cytochrome *c* oxidase subunit 2-NADH dehydrogenase subunit 1 gene region
*cytb*: cytochrome b
DTU: Discrete typing unit
gGAPDH: glyceraldehyde-3-phosphate dehydrogenase
*GPI*: Glucose-6-phosphate isomerase
ITS: Internal transcribed spacer
K2P: Kimura 2-parameter
LIT: Liver infusion tryptose
ML: Maximum likelihood
MLST: Multilocus sequencing typing
MP: Maximum parsimony
mPTP: multi-rate Poisson Tree Processes
NJ: Neighbor-joining
NNN: Novy-McNeal-Nicole medium
PTP: single-rate Poisson Tree Processes
SSU rDNA: Small subunit ribosomal DNA
V7 V8: Variable regions 7 and 8
