# Supplementary figures and images for: Cytochrome c oxidase subunit 1 gene as a DNA barcode for discriminating Trypanosoma cruzi DTUs and closely related species
Source: Parasit Vectors. 2017 Oct 16;10:488. doi: 10.1186/s13071-017-2457-1 (PMC5644147; doi:10.1186/s13071-017-2457-1)

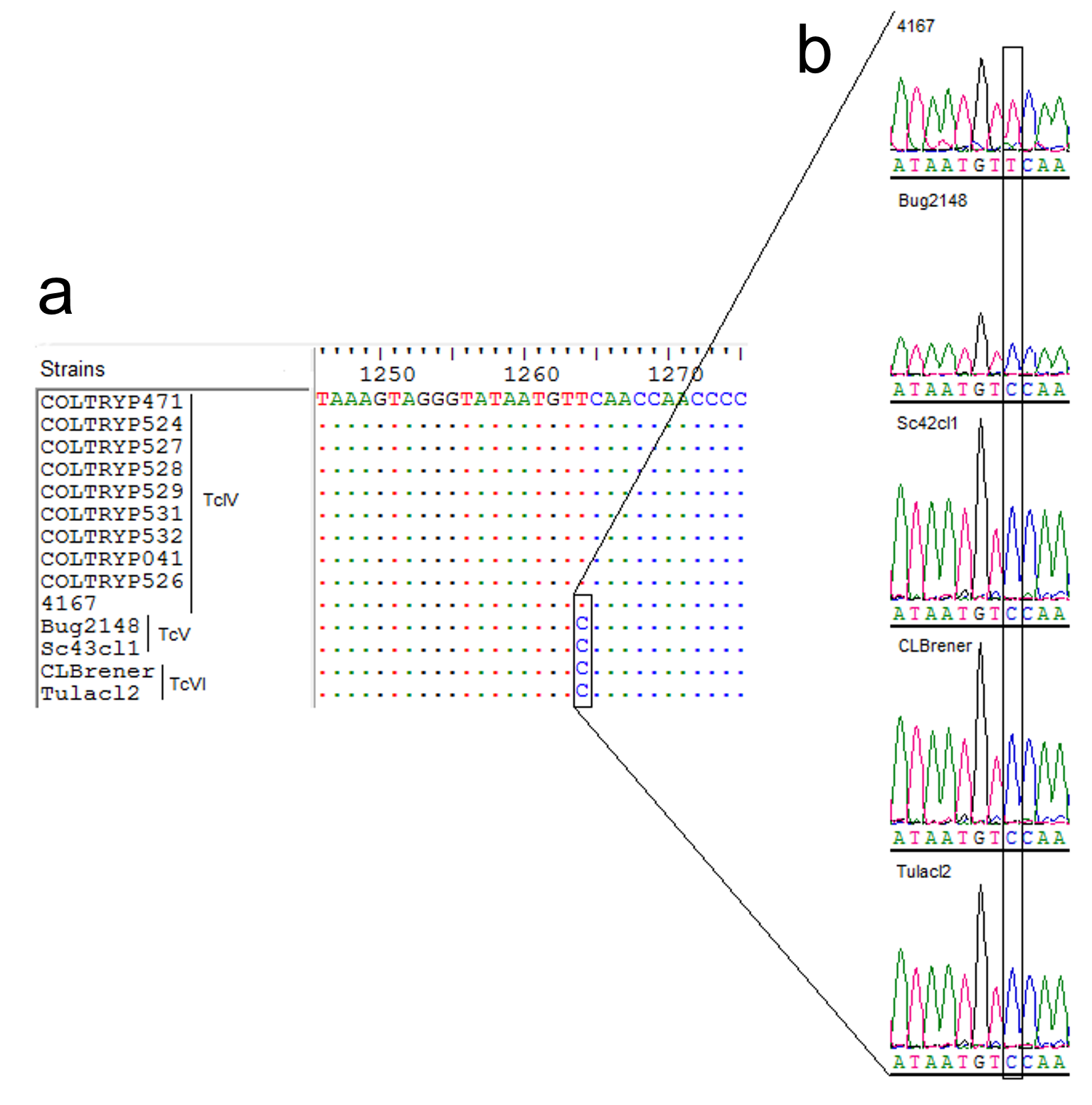

Supplement: Supplementary file 1 — Comparison between TcIV and TcV/TcVI nucleotide sequences generated with the cox1 barcode. a Alignment of TcIV sequences with TcV and TcVI shows one single nucleotide polymorphism differentiating TcIV samples from the hybrids. b Electropherogram confirms the presence of a T (thymine) in TcIV in the same position, showing a C (cytosine) in TcV and TcVI sequences. (TIFF 724 kb) [file 13071_2017_2457_MOESM1_ESM.tif]

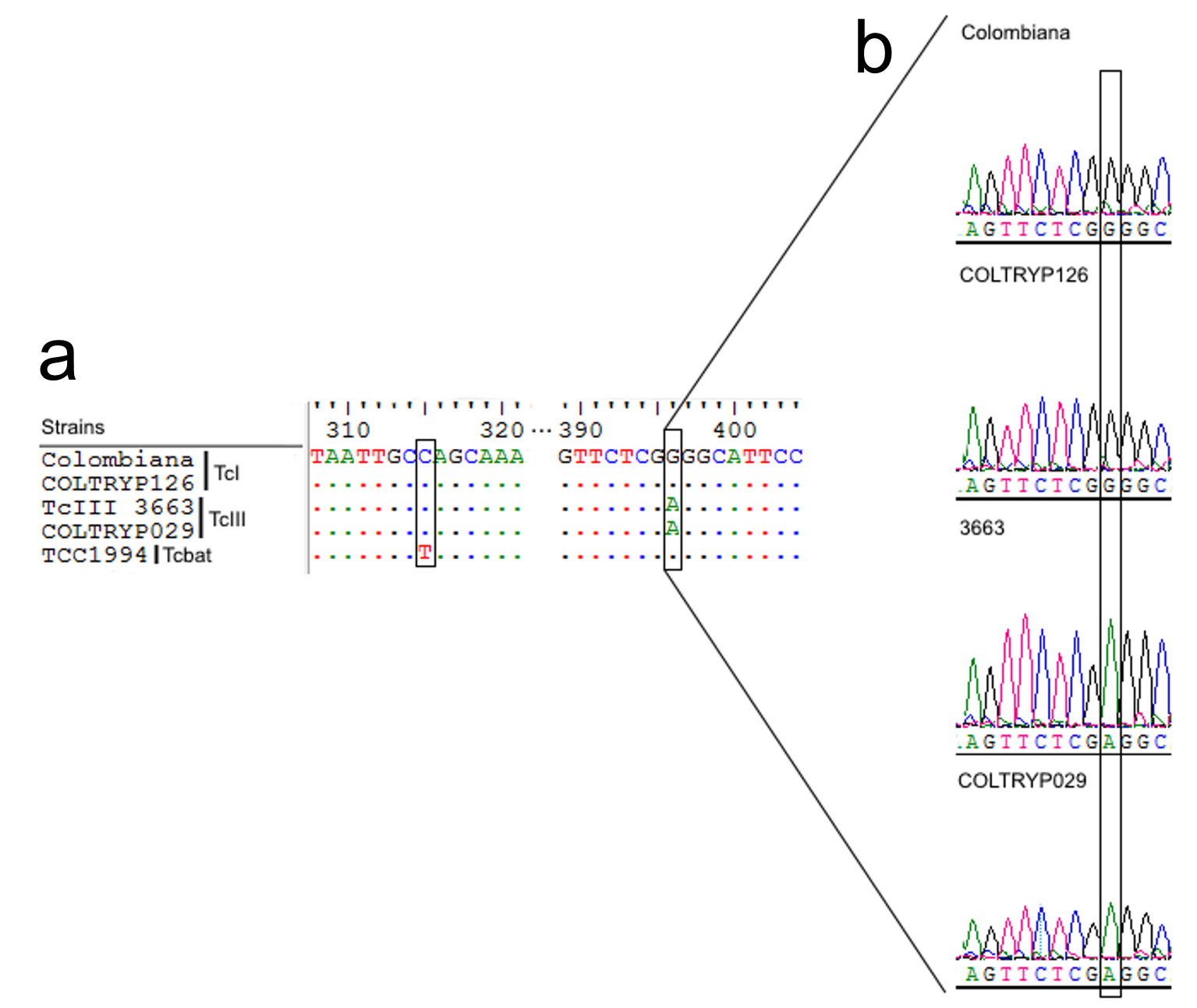

Supplement: Supplementary file 2 — Comparison between TcI, Tcbat and TcIII nucleotide sequences generated with GPI. a Sequence alignment shows one single nucleotide polymorphism differentiating TcI from Tcbat and one polymorphism separating TcI from TcIII sequences. b Electropherogram confirms the presence of A (adenine) in TcIII sequences in the same position, showing a G (guanine) in TcI sequences. The Tcbat sequence was retrieved from GenBank and the electropherogram is not publicly available. (TIFF 527 kb) [file 13071_2017_2457_MOESM2_ESM.tif]
