# Supplementary material for: Cytochrome c oxidase subunit 1 gene as a DNA barcode for discriminating Trypanosoma cruzi DTUs and closely related species
Source: Parasit Vectors. 2017 Oct 16;10:488. doi: 10.1186/s13071-017-2457-1 (PMC5644147; doi:10.1186/s13071-017-2457-1)
Supplement: Supplementary file 3 — Number of haplotypes, nucleotide diversity and haplotype diversity of sequences generated with cox1 and GPI. (DOCX 12 kb) [file 13071_2017_2457_MOESM3_ESM.docx]

| **Additional file 3: Table S1** Number of haplotypes, nucleotide diversity and haplotype diversity of sequences generated with COI and GPI. | | | | |
| --- | --- | --- | --- | --- |
| Group | N | Haplotype | π ± SD | Hd ± SD |
| COI |  |  |  |  |
| TcI | 31 | 8 | 0.01276 ± 0.00074 | 0.832 ± 0.035 |
| TcII | 14 | 3 | 0.00143 ± 0.00038 | 0.538 ± 0.115 |
| TcIII | 4 | 3 | 0.00485 ± 0.00192 | 0.833 ± 0.222 |
| TcIV | 10 | 1 | 0.00000 ± 0.00000 | 0.000 ± 0.000 |
| TcV/TcVI | 4 | 1 | 0.00000 ± 0.00000 | 0.000 ± 0.000 |
| *T. c. marinkellei* | 7 | 3 | 0.02493 ± 0.00872 | 0.667 ± 0.160 |
| *T. dionisii* | 5 | 1 | 0.00000 ± 0.00000 | 0.000 ± 0.000 |
| *T. rangeli* | 2 | 2 | 0.05593 ± 0.02796 | 1.000 ± 0.500 |
| Group | N | Haplotype | π ± SD | Hd ± SD |
| GPI |  |  |  |  |
| TcI | 31 | 2 | 0.00026 ± 0.00014 | 0.094 ± 0.049 |
| TcII | 14 | 2 | 0.00089 ± 0.00016 | 0.423 ± 0.076 |
| TcIII | 5 | 2 | 0.00096 ± 0.00017 | 0.533 ± 0.095 |
| TcIV | 11 | 2 | 0.00040 ± 0.00023 | 0.173 ± 0.101 |
| TcV/TcVI | 4 | 1 | 0.00000 ± 0.00000 | 0.000 ± 0.000 |
| *T. c. marinkellei* | 7 | 5 | 0.00338 ± 0.00080 | 0.736 ± 0.107 |
| *T. dionisii* | 5 | 1 | 0.00000 ± 0.00000 | 0.000 ± 0.000 |
| *T. rangeli* | 2 | 2 | 0.00585 ± 0.00179 | 0.667 ± 0.204 |
| N = total number of sequences used in the present study and from GenBank; π = nucleotide diversity; Hd = haplotype diversity, SD = standard deviation. | | | | |
| *GPI haplotypes were inferred after haplotype reconstruction with the algorithm PHASE | | | | |
| ^a^ TcV/TcVI sequences have one haplotype for each allele. | | | | |
