# Supplementary material for: Cytochrome c oxidase subunit 1 gene as a DNA barcode for discriminating Trypanosoma cruzi DTUs and closely related species
Source: Parasit Vectors. 2017 Oct 16;10:488. doi: 10.1186/s13071-017-2457-1 (PMC5644147; doi:10.1186/s13071-017-2457-1)
Supplement: Supplementary file 4 — Inter- and intraspecific genetic distance based on cox1 sequences. (DOCX 14 kb) [file 13071_2017_2457_MOESM4_ESM.docx]

| **Additional file 4: Table S2** Inter- and intraspecific genetic distance based on COI sequences. | | | |
| --- | --- | --- | --- |
| **Groups** | **Min** | **Max** | **Mean** |
| **Inter-DTU** |  |  |  |
| Tcbat X TcI | 0.054 | 0.064 | 0.060 |
| Tcbat X TcII | 0.099 | 0.105 | 0.103 |
| Tcbat X TcIII | 0.100 | 0.105 | 0.102 |
| Tcbat X TcIV | 0.105 | 0.105 | 0.105 |
| Tcbat X TcV/TcVI | 0.108 | 0.108 | 0.108 |
| TcI X TcII | 0.094 | 0.118 | 0.109 |
| TcI X TcIII | 0.100 | 0.113 | 0.107 |
| TcI X TcIV | 0.105 | 0.113 | 0.110 |
| TcI X TcV/TcVI | 0.105 | 0.116 | 0.112 |
| TcII X TcIII | 0.105 | 0.114 | 0.111 |
| TcII X TcIV | 0.119 | 0.124 | 0.123 |
| TcII X TcV/TcVI | 0.122 | 0.127 | 0.126 |
| TcIII X TcIV | 0.020 | 0.025 | 0.022 |
| TcIII X TcV/TcVI | 0.023 | 0.027 | 0.025 |
| TcIV X TcV/TcVI | 0.002 | 0.002 | 0.002 |
|  |  |  |  |
| **Interspecific** |  |  |  |
| *T. cruzi* X *T.c.marinkellei* | 0.101 | 0.153 | 0.130 |
| *T.cruzi* X *T. dionisii* | 0.130 | 0.160 | 0.150 |
| *T. cruzi* X *T. rangeli* | 0.147 | 0.213 | 0.182 |
| *T.c.marinkellei* X *T. dionisii* | 0.135 | 0.163 | 0.156 |
| *T.c.marinkellei* X *T. rangeli* | 0.157 | 0.198 | 0.178 |
| *T. dionisii* X *T. rangeli* | 0.151 | 0.188 | 0.169 |
| **Intra-DTU** |  |  |  |
| Tcbat | NC | NC | NC |
| TcI | 0.000 | 0.028 | 0.013 |
| TcII | 0.000 | 0.004 | 0.001 |
| TcIII | 0.000 | 0.009 | 0.005 |
| TcIV | 0.000 | 0.000 | 0.000 |
| TcV/TcVI | 0.000 | 0.000 | 0.000 |
|  |  |  |  |
| **Intraspecific** |  |  |  |
| *T. cruzi* | 0.000 | 0.127 | 0.076 |
| *T.c.marinkellei* | 0.000 | 0.062 | 0.026 |
| *T. dionisii* | 0.000 | 0.000 | 0.000 |
| *T. rangeli* | NC | NC | 0.059 |
| NC = not calculated. Low number of sequences available for calculation. | | | |
| Top: genetic distance between *Trypanosoma* species *T. cruzi*, *T. c. marinkellei*, *T. dionisi*i and *T. rangeli* | | | |
| Bottom: genetic distance within *T. cruzi* subpopulations, *T. c. marinkellei*, *T. dionisii* and *T. rangeli* | | | |
