# Supplementary material for: Cytochrome c oxidase subunit 1 gene as a DNA barcode for discriminating Trypanosoma cruzi DTUs and closely related species
Source: Parasit Vectors. 2017 Oct 16;10:488. doi: 10.1186/s13071-017-2457-1 (PMC5644147; doi:10.1186/s13071-017-2457-1)
Supplement: Supplementary file 5 — Inter- and intraspecific genetic distance based on GPI sequences. (DOCX 15 kb) [file 13071_2017_2457_MOESM5_ESM.docx]

| **Additional file 5: Table S3** Inter- and intraspecific genetic distance based on GPI sequences. | | | |
| --- | --- | --- | --- |
| **Groups** | **Min** | **Max** | **Mean** |
| **Inter-DTU** |  |  |  |
| Tcbat X TcI | 0.002 | 0.005 | 0.003 |
| Tcbat X TcII | 0.025 | 0.028 | 0.027 |
| Tcbat X TcIII | 0.005 | 0.005 | 0.005 |
| Tcbat X TcIV | 0.015 | 0.017 | 0.015 |
| Tcbat X TcV/TcVI | 0.005 | 0.025 | 0.015 |
| TcI X TcII | 0.022 | 0.028 | 0.024 |
| TcI X TcIII | 0.002 | 0.005 | 0.003 |
| TcI X TcIV | 0.012 | 0.015 | 0.013 |
| TcI X TcV/TcVI | 0.002 | 0.022 | 0.013 |
| TcII X TcIII | 0.025 | 0.028 | 0.027 |
| TcII X TcIV | 0.027 | 0.033 | 0.029 |
| TcII X TcV/TcVI | 0.000 | 0.028 | 0.014 |
| TcIII X TcIV | 0.015 | 0.017 | 0.015 |
| TcIII X TcV/TcVI | 0.000 | 0.025 | 0.012 |
| TcIV X TcV/TcVI | 0.015 | 0.030 | 0.021 |
|  |  |  |  |
| **Interspecific** |  |  |  |
| *T. cruzi* X *T.c.marinkellei* | 0.028 | 0.046 | 0.036 |
| *T.cruzi* X *T. dionisii* | 0.071 | 0.087 | 0.078 |
| *T. cruzi* X *T. rangeli* | 0.129 | 0.145 | 0.137 |
| *T.c.marinkellei* X *T. dionisii* | 0.056 | 0.064 | 0.059 |
| *T.c.marinkellei* X *T. rangeli* | 0.132 | 0.142 | 0.138 |
| *T. dionisii* X *T. rangeli* | 0.127 | 0.130 | 0.128 |
| **Intra-DTU** |  |  |  |
| Tcbat | NC | NC | NC |
| TcI | 0.000 | 0.002 | 0.000 |
| TcII | 0.000 | 0.002 | 0.001 |
| TcIII | 0.000 | 0.000 | 0.000 |
| TcIV | 0.000 | 0.002 | 0.000 |
| TcV/TcVI | 0.000 | 0.025 | 0.010 |
|  |  |  |  |
| **Intraspecific** |  |  |  |
| *T. cruzi* | 0.000 | 0.030 | 0.013 |
| *T.c.marinkellei* | 0.000 | 0.010 | 0.000 |
| *T. dionisii* | 0.000 | 0.000 | 0.000 |
| *T. rangeli* | NC | NC | 0.007 |
| NC = not calculated. Low number of sequences available for calculation | | | |
| Top: genetic distance between *Trypanosoma* species *T. cruzi*, *T. c. marinkellei*, *T. dionisi*i and *T. rangeli* | | | |
| Bottom: genetic distance within *T. cruzi* subpopulations, *T. c. marinkellei*, *T. dionisii* and *T. rangeli*. | | | |
