# Supplementary material for: Cytochrome c oxidase subunit 1 gene as a DNA barcode for discriminating Trypanosoma cruzi DTUs and closely related species
Source: Parasit Vectors. 2017 Oct 16;10:488. doi: 10.1186/s13071-017-2457-1 (PMC5644147; doi:10.1186/s13071-017-2457-1)
Supplement: Supplementary file 6 — Cox1 sequences partition into groups inferred with ABGD, based on Kimura 2-parameters. (DOCX 14 kb) [file 13071_2017_2457_MOESM6_ESM.docx]

| **Additional file 6: Table S4** COI sequences partition into groups inferred with ABGD, based on Kimura 2-parameters. | | | |
| --- | --- | --- | --- |
| Groups | Nº  of sequences | Sequence code | DTU |
| *T. cruzi* |  |  |  |
| Group 1 | 1 | TCC1994 | Tcbat |
| Group 2 | 5 | COLTRYP115,COLTRYP468, COLTRYP018, COLTRYP003, COLTRYP084 | TcI |
| Group 3 | 5 | COLTRYP126, COLTRYP339, Silvio, COLTRYP053, COLTRYP055 | TcI |
| Group 4 | 1 | COLTRYP036 | TcI |
| Group 5 | 9 | COLTRYP128, COLTRYP356, COLTRYP368, Ep88115, COLTRYP048, COLTRYP103, COLTRYP038, COLTRYP039, COLTRYP087 | TcI |
| Group 6 | 7 | COLTRYP136, COLTRYP224, Ep88127, Ep88132, Ep88135, Dm28c, COLTRYP042 | TcI |
| Group 7 | 1 | COLTRYP220 | TcI |
| Group 8 | 2 | COLTRYP305, COLTRYP362 | TcI |
| Group 9 | 1 | Colombiana | TcI |
| Group 10 | 14 | COLTRYP043, COLTRYP062, COLTRYP063, COLTRYP072, COLTRYP081, COLTRYP099, Ep88121, Ep88130, Y, Esmeraldo, COLTRYP006, COLTRYP021, COLTRYP061, COLTRYP121 | TcII |
| Group 11 | 4 | COLTRYP029, COLTRYP113, COLTRYP370, 3663 | TcIII |
| Group 12 | 10 | 4167, COLTRYP471, COLTRYP524, COLTRYP527, COLTRYP528, COLTRYP529, COLTRYP531, COLTRYP532, COLTRYP041, COLTRYP526 | TcIV |
| Group 13 | 5 | Bug2148, CLBrener, Sc43, Tulacl2, CLBrener (GenBank) | TcV/TcVI |
| *T. c. marinkellei* |  |  |  |
| Group 1 | 4 | COLTRYP107, B7, COLTRYP117, COLTRYP143 |  |
| Group 2 | 2 | COLTRYP576, COLTRYP577 |  |
| Group 3 | 1 | TCC344 |  |
| *T. dionisii* |  |  |  |
| Group 1 | 5 | COLTRYP596,COLTRYP598, COLTRYP621, COLTRYP622, COLTRYP623 |  |
| *T. rangeli* |  |  |  |
| Group 1 | 1 | R1625 |  |
| Group 2 | 1 | SC58 |  |
