# Supplementary material for: Cytochrome c oxidase subunit 1 gene as a DNA barcode for discriminating Trypanosoma cruzi DTUs and closely related species
Source: Parasit Vectors. 2017 Oct 16;10:488. doi: 10.1186/s13071-017-2457-1 (PMC5644147; doi:10.1186/s13071-017-2457-1)
Supplement: Supplementary file 7 — GPI sequences partition into groups inferred with ABGD, based on Kimura 2-parameters. (DOCX 14 kb) [file 13071_2017_2457_MOESM7_ESM.docx]

| **Additional file 7: Table S5** GPI sequences partition into groups inferred with ABGD, based on Kimura 2-parameters. | | | | |  |
| --- | --- | --- | --- | --- | --- |
| Groups | Nº  of sequences | Sequence code | DTU | |  |
| *T. cruzi* |  |  |  | |  |
| Group 1 | 1 | TCC1994 | Tcbat | |  |
| Group 2 | 31 | OPS21cl11, COLTRYP115, COLTRYP126, COLTRYP128, COLTRYP136, COLTRYP220, COLTRYP224, COLTRYP305, COLTRYP339, COLTRYP356, COLTRYP362, COLTRYP368, COLTRYP468, Ep88115, Ep88127, Ep88132, Ep88135, Colombiana, Dm28c, COLTRYP048, COLTRYP018, COLTRYP103, COLTRYP053, COLTRYP003, COLTRYP036, COLTRYP038, COLTRYP039, COLTRYP042, COLTRYP055, COLTRYP084, COLTRYP087 | TcI | | |
| Group 3 | 10 | COLTRYP043, COLTRYP062, COLTRYP063, COLTRYP072, COLTRYP081, COLTRYP099, Ep88130, Y, COLTRYP061, Ep88121 | TcII |  |  |
| Group 4 | 10 | COLTRYP121, COLTRYP006, COLTRYP021, Tu18cl2, CLBrener allele1(GenBank), Bug2148 allele1(GenBank), Sc43 allele1, Tulacl2 allele1, Bug2148 allele1, CLBrener allele1 | TcII/TcV/TcVI |  |  |
| Group 5 | 11 | COLTRYP029, COLTRYP113, COLTRYP370, 3663, M6241cl6, CLBrener allele2(GenBank), Bug2148 allele2(GenBank), Sc43 allele2, Tulacl2 allele2, Bug2148 allele2, CLBrener allele2 | TcIII/TcV/TcVI |  |  |
| Group 6 | 10 | 4167, COLTRYP471, COLTRYP524, COLTRYP527, COLTRYP528, COLTRYP529, COLTRYP531, COLTRYP532, COLTRYP041, COLTRYP526 | TcIV |  |  |
| Group 7 | 1 | CANIIIcl1 | TcIV |  |  |
| *T. c. marinkellei* |  |  |  |  |  |
| Group 1 | 4 | COLTRYP107, B7, COLTRYP117, COLTRYP143 |  |  |  |
| Group 2 | 2 | COLTRYP576 |  |  |  |
| Group 3 |  | COLTRYP577 |  |  |  |
| Group 4 | 1 | TCC344 |  |  |  |
| *T. dionisii* |  |  |  |  |  |
| Group 1 | 5 | COLTRYP596,COLTRYP598, COLTRYP621, COLTRYP622, COLTRYP623 |  |  |  |
| *T. rangeli* |  |  |  |  |  |
| Group 1 | 1 | R1625 |  |  |  |
| Group 2 | 1 | RGB |  |  |  |
